# The Clinical Utility of 3D Electroanatomical Mapping for Atrial Fibrillation Ablation by Pulsed Field Ablation

**DOI:** 10.1002/joa3.70234

**Published:** 2025-11-27

**Authors:** Robert N. Kerley, David Keane

**Affiliations:** ^1^ Department of Cardiology St. Vincent's University Hospital Dublin Ireland; ^2^ Department of Cardiology Blackrock Clinic Dublin Ireland

**Keywords:** atrial fibrillation, electroanatomical mapping, pulsed field ablation

## Abstract

**Background:**

Pulsed Field Ablation (PFA) is a tissue‐selective ablation energy source that has been introduced recently for atrial fibrillation (AF) ablation. Data on the use of 3D electroanatomic mapping (EAM) is limited in AF ablation by PFA with many centers electing to omit it.

**Objective:**

This study sought to investigate the utility of high‐density 3D EAM using PFA for AF ablation.

**Methods:**

Seventy‐four patients with symptomatic AF underwent PFA‐based pulmonary vein isolation (PVI). Additional ablation, including left atrial posterior wall (LAPW) and mitral‐isthmus (MI) ablation was performed in a subset of patients. The primary efficacy endpoint was freedom from atrial arrhythmia at 12 months. The primary safety endpoint was freedom from a composite of serious procedure‐ and device‐related adverse events.

**Results:**

In 74 patients, 3D EAM post‐PFA showed early PV reconnection in 7/74 cases, (9% cases; 289/296 PVs, 2.4% PVs), most commonly in the right superior PV (6/7, 85.7%). The LAPW reconnected in 3/55 cases (5.5%), while the MI line reconnected in 6/14 cases (30%), more commonly with an anterior approach compared to a posterior (57% vs. 15%). The procedure time was 88.3 ± 40.7 min and fluoroscopic time was 12.1 ± 8.0 min. At 1 year, estimated freedom from atrial arrhythmia was 78.4% (95% CI, 70.1 to 88.7). There was 1 case of pericardial tamponade.

**Conclusion:**

Our results suggest that although there is a low incidence, early PV reconnection can still occur using PFA for PVI. Overall 3D EAM retains clinical value in AF ablation by PFA.

## Graphical abstract

Systematic high‐density 3D electroanatomical remapping during pulsed field ablation detected residual conduction in 9% of cases, predominantly in the right superior pulmonary vein. Detailed voltage analysis demonstrated sharp 1 mm lesion borders, highlighting the value of 3D mapping for assessing lesion integrity and procedural endpoints in pulsed field ablation.

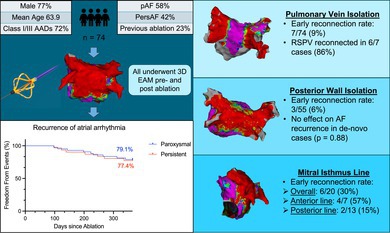

## 1 Introduction

The treatment of atrial fibrillation by catheter ablation has expanded greatly in the last two decades, now considered a first‐line treatment for select patients in international guidelines [1]. Thermal catheter ablation is associated with several potential complications including esophageal injury, pulmonary vein (PV) stenosis, and phrenic nerve injury [2]. Pulsed field ablation (PFA) is a recently approved form of ablation energy that can be used to perform AF catheter ablation via a mechanism called irreversible electroporation [3]. This method involves delivering an electrical field of energy to cardiac cells, which increases their membrane permeability leading to cell death via protein denaturation or tissue scaffolding damage [3]. The threshold for irreversible electroporation varies among cell types, and we have previously reported differences in susceptibility in vitro for myocytes, neurons, adipocytes, and esophageal cells [3, 4, 5]. Clinical studies suggest that PV isolation (PVI) by PFA is safe, reliable, and efficient [6, 7, 8]. Here we present our data from the first twelve months of PFA at our center.

## 2 Methods

### 2.1 Patient Selection

The study was a single‐center prospective cohort study of patients referred with paroxysmal and persistent AF ablation. Patients were referred for AF ablation as a first‐line treatment or more commonly following an intolerance or refractoriness to anti‐arrhythmic drugs (AADs). Inclusion was based on referral to our center from March 2022 to March 2023. Choice of PFA‐PVI was based on the suspicion of having other types of atrial arrhythmia other than AF in which case patients were deemed unsuitable for PFA‐PVI. Data on all patients were collected on our local PFA AF database prospectively. Variables collected included date of procedure, age, sex, type of AF, left ventricular ejection fraction (LVEF), skin‐to‐skin procedure time, fluoroscopic time, ablation strategy, and follow‐up Holter result. Institutional review board ethics approval was sought and attained. The analysis was conducted according to the Declaration of Helsinki guidelines.

### 2.2 Procedural Outline

A computed tomography (CT) of the left atrium (LA) was performed in the days prior to AF ablation in all cases. All were performed with patients intubated under general anesthesia on uninterrupted direct oral anticoagulants (DOAC), and specifically, all patients were given their DOAC on the morning of the procedure. Two right femoral vein punctures were obtained with or without ultrasound in accordance with physical findings. A deflectable 20‐pole catheter was placed in the right atrium and the coronary sinus. Transseptal puncture was performed under dual‐plane fluoroscopic and radiographic contrast guidance by use of an 8F SL‐1 sheath and a BRK‐0 needle (manually modified based on the degree of right atrial dilatation). Upon access to the LA, heparin (100 IU/kg) was given. The SL‐1 sheath was then exchanged for a 16.8‐Fr deflectable transseptal sheath (Faradrive, Boston Scientific) via an over‐the‐wire exchange. Through the deflectable sheath, a multispline multielectrode mapping catheter (Octaray, Biosense Webster) was introduced to the LA and a 3D electroanatomical map was created (Carto, Biosense Webster). Following this, the Octaray catheter was replaced by a five‐spline 12F PFA catheter (Farawave, Boston Scientific) using the same deflectable sheath (Farawave) in the LA.

Given the large electrode size and the very large interelectrode spacing of the Farawave catheter, we selected two parallel filter settings for the display of the electrograms from the Farawave catheter:

1. Standard filter settings of 30–500 Hz were used, which included a significant degree of far‐field electrograms from the ventricle as well as the left atrial appendage.
2. Higher filter settings of 100–500 Hz bandwidth in order to reduce the proportion of far‐field electrograms.

The Farawave catheter can be configured into different shapes (basket and flower configurations) for energy delivery. Our protocol involves at least 8 applications (4 baskets, 4 flowers), where 1 application is defined as 4 bipolar pulse trains 100–200 ms at 2.0 kV. The catheter is slightly rotated (> 30 degrees) between pairs of applications to ensure circumferential PV ostial and antral coverage. After 8 applications, all PV ostia were checked for isolation using the basket configuration of the catheter. In patients with persistent AF, additional ablation lesions were performed at the discretion of the operator. This included left atrial posterior wall (LAPW) isolation and mitral isthmus (MI) ablation, which were performed in the flower configuration with the guidewire retracted. In accordance with the distribution of atrial fibrosis on the baseline left atrial map, mitral isthmus lines were either anteroseptal (from the anterior mitral annulus to the anterior aspect of the right superior pulmonary vein antrum taking care to avoid further extension up to the LA roof line) or posterolateral (from the posterolateral mitral annulus to the inferior aspect of the left inferior pulmonary vein antrum). After PVI and possible additional lesions, the PFA catheter was removed, and the multispline multielectrode mapping catheter (Octaray) was reintroduced for repeat mapping.

### 2.3 Voltage Mapping and Reconnection Criteria

Voltage maps were created using a standardized bipolar voltage range of 0.05–0.5 mV to define scar tissue (displayed in red), with tissue > 0.5 mV considered healthy myocardium (displayed in purple/violet). Custom voltage settings of 0.05–0.5 mV were used to enhance visualization of scar borders during analysis. Acute PV reconnection was defined as the identification of PV potentials on the pulmonary vein side of the ablation line, or the presence of electrical conduction across the wide area circumferential ablation line demonstrated by activation mapping. Similarly, LAPW reconnection was defined as electrical conduction across the posterior wall ablation line, and mitral isthmus (MI) reconnection was defined as the absence of bidirectional conduction block across the MI line, confirmed by differential pacing maneuvers.

### 2.4 Pulsed Field Ablation Scar Margin Analysis

We present a novel method for assessing the scar border margins on the 3D EAM. In all cases, the scar border line on the 3D EAM was manually split into 4 roughly equal segments. The patient's right PV wide area of circumferential ablation (WACA), and in cases where it was performed the LAPW line was used. The right PV WACA and LAPW lines were chosen to avoid LA appendage far‐field signal interference. In each segment, 5 points have been taken across the scar border in each of the 4 segments. An average for each of these 5 points across the whole scar border line is then taken as the gradient from scar to healthy tissue. A custom voltage was set to 0.05–0.5 mV to enhance the scar border for easier identification of gradient in all cases.

### 2.5 Follow‐Up and Endpoints

The primary endpoint was freedom from atrial arrhythmia recurrence, defined as any episode of atrial fibrillation (AF), atrial flutter (AFL), or atrial tachycardia (AT) lasting ≥ 30 s, documented by 12‐lead ECG or 24‐h Holter monitoring, occurring after a 90‐day blanking period following ablation. Secondary endpoints included the need for repeat ablation, direct current cardioversion, or anti‐arrhythmic drug escalation during the 12‐month follow‐up period. The 90‐day blanking period was implemented to allow for lesion maturation and to exclude early recurrences that may not represent true procedural failure. Follow‐up included office visits at 3, 6, and 12 months, each with 12‐lead ECG and 24‐h Holter monitoring. The primary safety endpoint was freedom from a composite of serious procedure‐ and device‐related adverse events. All acute or chronic complications of cardiac tamponade, thromboembolism, stroke, transient ischaemic attack, phrenic nerve paralysis, heart block, pericarditis, vascular complications requiring intervention or prolonged hospital stay, atrioesophageal fistula and death were registered.

### 2.6 Statistical Analysis

Clinical recurrence‐free survival analysis was calculated using the Kaplan‐Meier method. All primary analysis patients who underwent PFA were included in the analysis. Time 0 was defined as the date of ablation. For continuous variables, the mean ± standard deviation (SD) was used. For group comparisons, Student's *t*‐test (paired or unpaired) or the Mann‐Whitney *U* test for unpaired variables was used. Categorical data were described as percentages and frequencies; they were compared using Chi‐square or Fisher's exact testing. Statistical analysis was performed using GraphPad Prism 7.0 software and SPSS version 7.0.

## 3 Results

### 3.1 Baseline Characteristics and Follow Up Duration

A total of 74 patients, 43 with paroxysmal AF and 31 with persistent AF, underwent AF catheter ablation by PFA. Baseline characteristics are shown in Table 1. Patients were 63.9 ± 9.9 years, 77% male, 42% had hypertension, 11% had diabetes mellitus, and 16% had a history of heart failure. The mean LVEF was 55% ± 5% with a mean LA diameter of 4.3 ± 0.6 cm. Concomitant pharmacotherapy with a Class I or Class III AAD was used in 53 patients (71.6%). A total of 17 patients (23%) had a previous AF catheter ablation, 6 patients (8%) had a previous atrial flutter (AFL) ablation, and 1 patient (1%) had a Maze procedure.

**TABLE 1:** Patient characteristics.

Patient characteristics	*n* = 74
Age (years)	63.9 ± 9.9
Male (%)	57 (77.0)
Type of AF	
Paroxysmal AF (%)	43 (58.1)
Persistent AF (%)	31 (41.9)
LVEF	54.9 ± 5.1
LA diameter	4.3 ± 0.6
Previous catheter ablation	
AF ablation	17 (23.0)
AFL ablation	6 (8.1)
Cox Maze procedure	1 (1.4)
CHA_2_DS_2_VaSc score	3 (2–4)
Diabetes	8 (10.8)
Hypertension	42 (56.8)
Heart failure	12 (16.2)
Class I or III AADs	53 (71.6)

*Note:* Values are represented as median ± SD or *n* (%).

Abbreviations: AAD, anti‐arrhythmic drugs; AF, atrial fibrillation; AFL, atrial flutter; LA, left atrium; LVEF, left ventricular ejection fraction.

### 3.2 Procedural Characteristics

Baseline procedure characteristics are shown in Table 2. All procedures were performed under general anesthetic with a median procedure time of 88.3 ± 40.7 min and a median fluoroscopic time of 12.1 ± 8.0 min. All patients had 3D EAM of the LA pre and post ablation with a multi‐spline mapping catheter primarily in sinus rhythm (64%). A 31‐mm device was used in 57 patients (77%) and a 35‐mm device was used in 17 patients (23%) with a median number of PFA applications of 40.5 ± 8.2. A switch to radiofrequency ablation (RFA) from PFA occurred in 3 cases (4%).

**TABLE 2:** Procedural characteristics.

Procedure time	88.3 ± 40.7 min
Fluoroscopic time	12.1 ± 8.0 min
Mapping catheter used	74 (100)
Switch to RF ablation	3 (4.3)
Total PFA applications per patient	40.5 ± 8.2
PFA catheter size	
31 mm	57 (77.0)
35 mm	17 (23.0)
Rhythm during mapping	
Sinus rhythm	47 (63.5)
Atrial fibrillation	27 (36.5)

*Note:* Values are represented as median ± SD or *n* (%).

Abbreviations: PFA, pulsed field ablation; RF, radiofrequency.

### 3.3 Primary Endpoints

**FIGURE 1:**
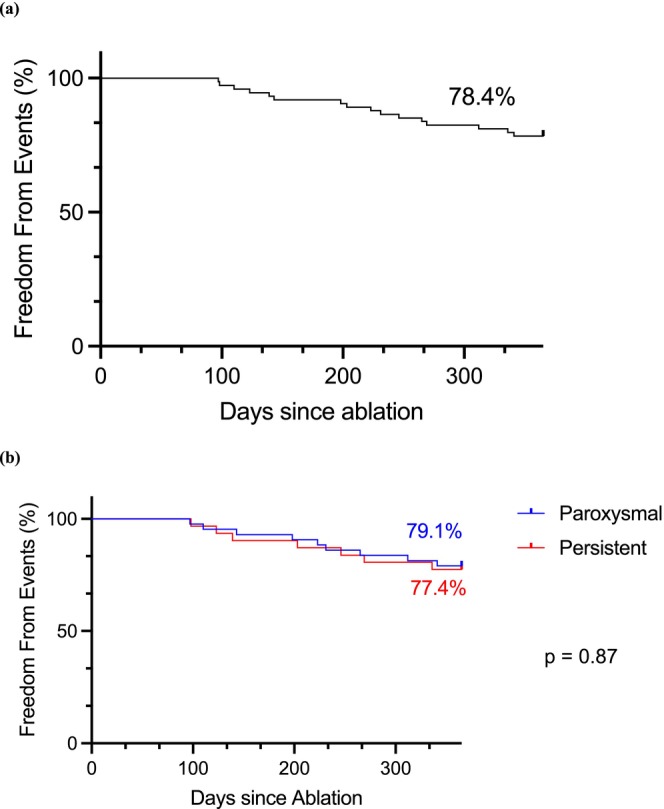
Treatment success at 12 months for paroxysmal and persistent atrial fibrillation. (a) Recurrence rate of atrial arrhythmia across the entire cohort 12 months post‐ablation. (b) There was no difference in atrial arrhythmia recurrence at 12 months between patients with persistent versus paroxysmal atrial fibrillation.

The primary endpoint, a composite endpoint of acute procedural failure, arrhythmia recurrence, repeat AF ablation, direct current cardioversion, or AAD escalation over 12 months post‐ablation, excluding a 90‐day blanking period is shown in Figure 1. PFA was shown to be effective at 1 year in 78.4% (95% CI, 70.1 to 88.7) overall, 79.1% (95% CI, 69.2% to 94.4%) for paroxysmal AF, and 77.4% (68.1% to 94.5%) for persistent AF. First recorded arrhythmia after the blanking period was atrial tachycardia (AT) in 2/16 (12.5%) and AF in 14/16 (87.5%). There was no significant difference between paroxysmal and persistent AF patients in terms of recurrence rates (79.1% vs. 77.4%, *p* = 0.87). Table 3 shows a comparison between baseline and procedural characteristics for patients with arrhythmia recurrence. Previous AF/AFL ablation was a significant risk factor for AF recurrence post PFA at 12 months with 12/16 patients (75%) in the recurrence group having a previous AF/AFL ablation versus 12/58 patients (20.7%) in the atrial arrhythmia‐free group (*p* < 0.001). Holter monitor recurrence rates were 6 cases (8.1%) at 3 months, 7 cases (9.4%, cumulative 17.5%) at 6 months and 3 cases (4.1%, cumulative 21.4%) at 12 months.

**TABLE 3:** Comparison of baseline and procedural characteristics for patients with arrhythmia recurrence versus those without.

	Recurrence (*n* = 16)	No recurrence (*n* = 58)	*p* value
Age (years)	67.2 ± 9.9	63.1 ± 9.1	0.55
Male (%)	81.3	75.9	0.65
Persistent AF	7 (43.8)	24 (41.4)	0.87
Previous ablation	12 (75.0)	12 (20.7)	< 0.001
Previous AF ablation	8 (50.0)	9 (15.5)	
Previous AFL ablation	3 (5.2)	3 (18.8)	
Previous MAZE	1 (6.3)	0	
LVEF	53 ± 5.7	56 ± 6.4	0.36
LA diameter	4.6 ± 1.2	4.2 ± 0.6	0.56
CHA_2_DS_2_VaSc score	3.1	3.4	0.79
Diabetes	2 (12.5)	6 (10.3)	0.67
Hypertension	9 (56.3)	35 (60.3)	0.45
Heart failure	3 (18.8)	9 (15.5)	0.27
Class I or III AADs	12 (75.0)	41 (70.7)	0.34
Procedure time	88.3 ± 40.7 min	88.3 ± 40.7 min	0.62
Fluoroscopic time	12.4 ± 10.2	12.0 ± 9.1	0.89
Switch to RF ablation	1 (6.3%)	2 (3.4%)	0.16
35 mm PFA catheter	5 (25.0%)	12 (20.7%)	0.56
Additional ablation			
Posterior wall isolation	11 (68.8)	44 (75.9)	0.56
MI ablation	6 (37.5)	14 (24.1)	0.29
Anterior MI line	2 (12.5)	5 (8.6)	0.56
Posterior MI line	4 (25.0)	9 (15.5)	0.56
CTI ablation	1 (6.3)	1 (1.7)	0.89
Termination of AF			0.159
With ablation	11 (68.8)	36 (62.1)	
With DCCV	3 (18.8)	18 (31.0)	
With RFA	2 (12.5)	1 (1.7)	
Pace termination	0	3 (5.2)	
Postmap PV reconnection	1 (6.3)	6 (10.3)	0.62
Postmap PW reconnection	1 (6.3)	3 (5.2)	0.74
Postmap MI reconnection	4 (25.0)	2 (3.4)	0.02

Abbreviations: AAD, anti‐arrhythmic drugs; AF, atrial fibrillation; AFL, atrial flutter; CTI, cavo‐tricuspid isthmus; DCCV, direct current cardioversion; LA, left atrium; MI, mitral isthmus; PFA, pulsed field ablation; PV, pulmonary vein; PW, posterior wall; RF, radiofrequency.

### 3.4 Primary Safety Endpoints

The acute and chronic complications are presented in Table 4. There was one cardiac tamponade in a case involving the switch from PFA to RFA ablation. There were no recorded complications in cases involving PFA alone. We did not observe temporary or permanent phrenic nerve palsy, vascular complications, ventricular arrhythmias, stroke or transient ischaemic attack, myocardial infarction, coronary infarction, coronary vasospasm, PV stenosis, atrioesophageal fistula, pulmonary oedema, or pericarditis. We observed no deaths related to the procedure. We commonly observed sinoatrial and atrioventricular block during the ablation of the right superior and inferior veins, which was effectively treated with acute or prophylactic intravenous atropine injection or ventricular pacing from the coronary sinus catheter.

**TABLE 4:** Primary safety outcomes.

Procedural characteristics	Total
Total	1 (1.4)
Death	0 (0)
Myocardial infarction	0 (0)
Coronary vasospasm	0 (0)
Diaphragmatic paralysis	0 (0)
Stroke or TIA	0 (0)
Other thromboembolism	0 (0)
Cardiac tamponade or perforation	1 (1.4)
Vascular complications	0 (0)
PV stenosis	0 (0)
Atrioesophageal fistula	0 (0)
Pericarditis	0 (0)
Pulmonary oedema	0 (0)

*Note:* Values are represented as *n* (%).

Abbreviations: PV, pulmonary vein; TIA, transient ischaemic attack.

### 3.5 3D Mapping, Pulmonary Vein Reconnection and Additional Ablation

**FIGURE 2:**
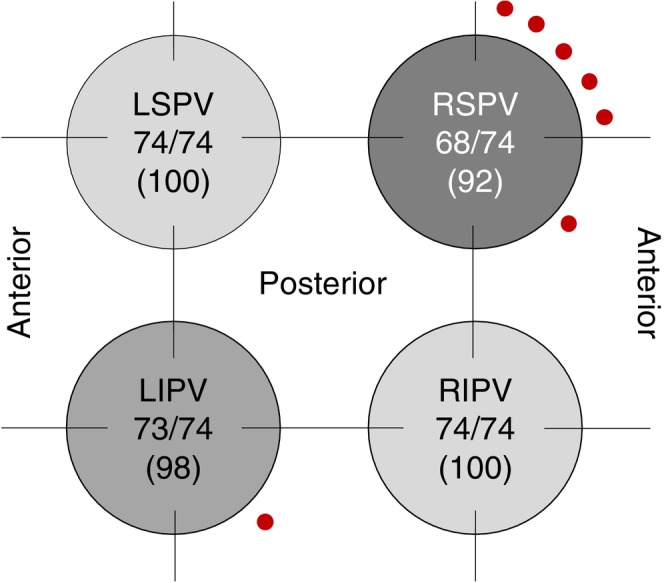
Pulmonary vein reconnection rate. The pulmonary vein reconnection rate was 2.4% 7/296 with the RSPV most commonly reconnecting. Most gaps were located in the anterior‐superior aspect of the RSPV ostium 5/6 (83.3%). LIPV, left inferior pulmonary vein; LSPV, left superior pulmonary vein; RIPV, right inferior pulmonary vein; RSPV, right superior pulmonary vein.

**FIGURE 3:**
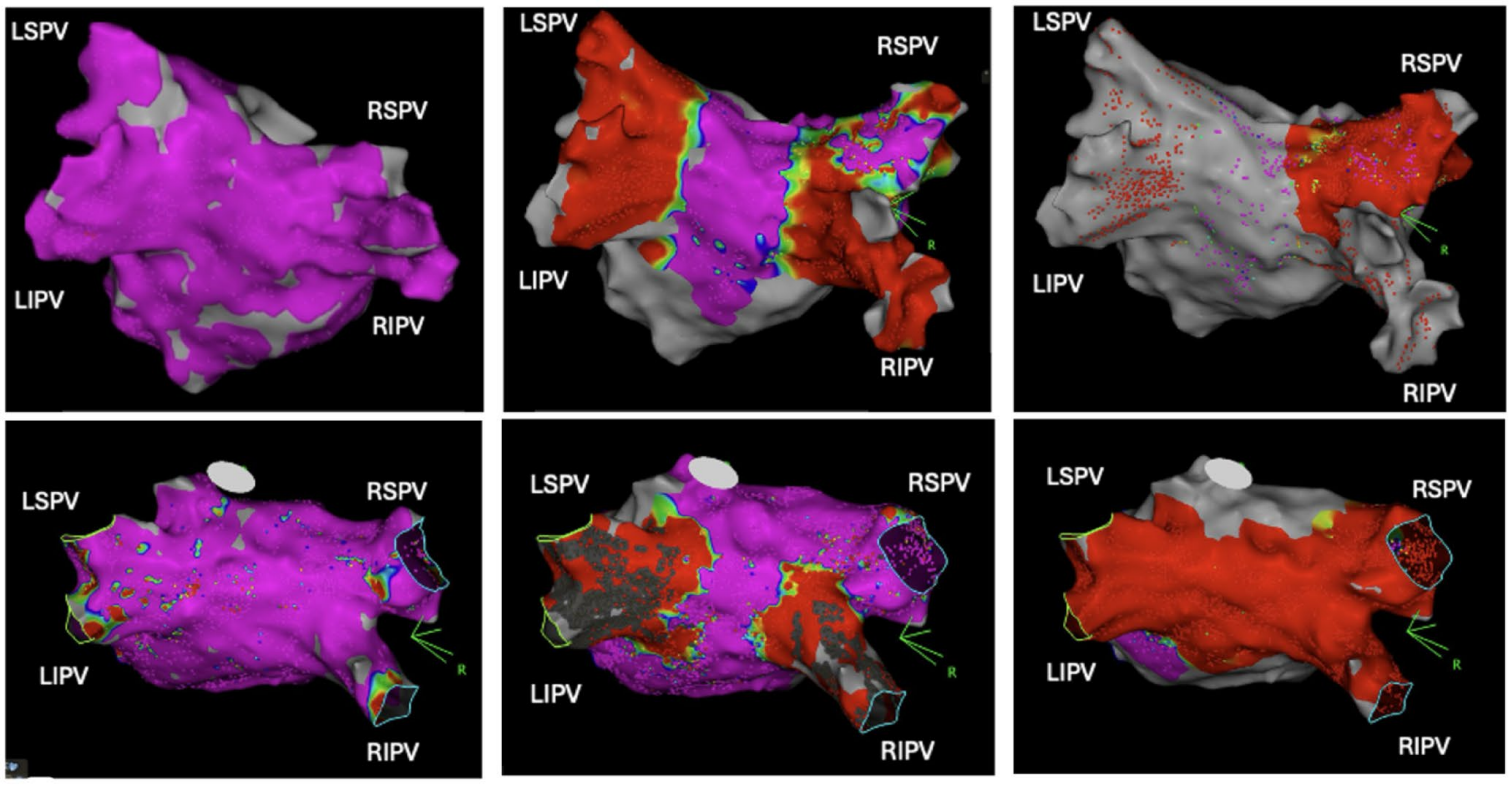
Pulmonary vein reconnections on high‐density 3D Electroanatomic Mapping. Leftward panel shows pre‐ablation 3D electroanatomic mapping. Middle panel shows post‐ablation 3D electroanatomic mapping. Rightward panel shows remapping post‐ablation of the acutely reconnected pulmonary vein.

All 74/74 patients received a pre‐ and post‐ablation high‐density 3D EAM. Mapped LA volume including PVs was 151 ± 41.6 mL with 7834 ± 3895 acquired mapping points. Post‐ablation high density 3D EAM revealed 7 early PV reconnections 7/296 (2.4% reconnection rate) in 7 patients (7/74 cases, 9.5%; LSPV: *n* = 0; LIPV: *n* = 1; RSPV: *n* = 6; RIPV: *n* = 0, Figure 2). Most gaps were in the anterior‐superior aspect of the PV ostium (Figures 2 and 3). A median of 4.0 ± 2.5 additional PFA applications with 2.0 kV were delivered to isolate the reconnected PVs. In all cases, the PVs were isolated at the end of the procedure as confirmed by high‐density 3D EAM.

**FIGURE 4:**
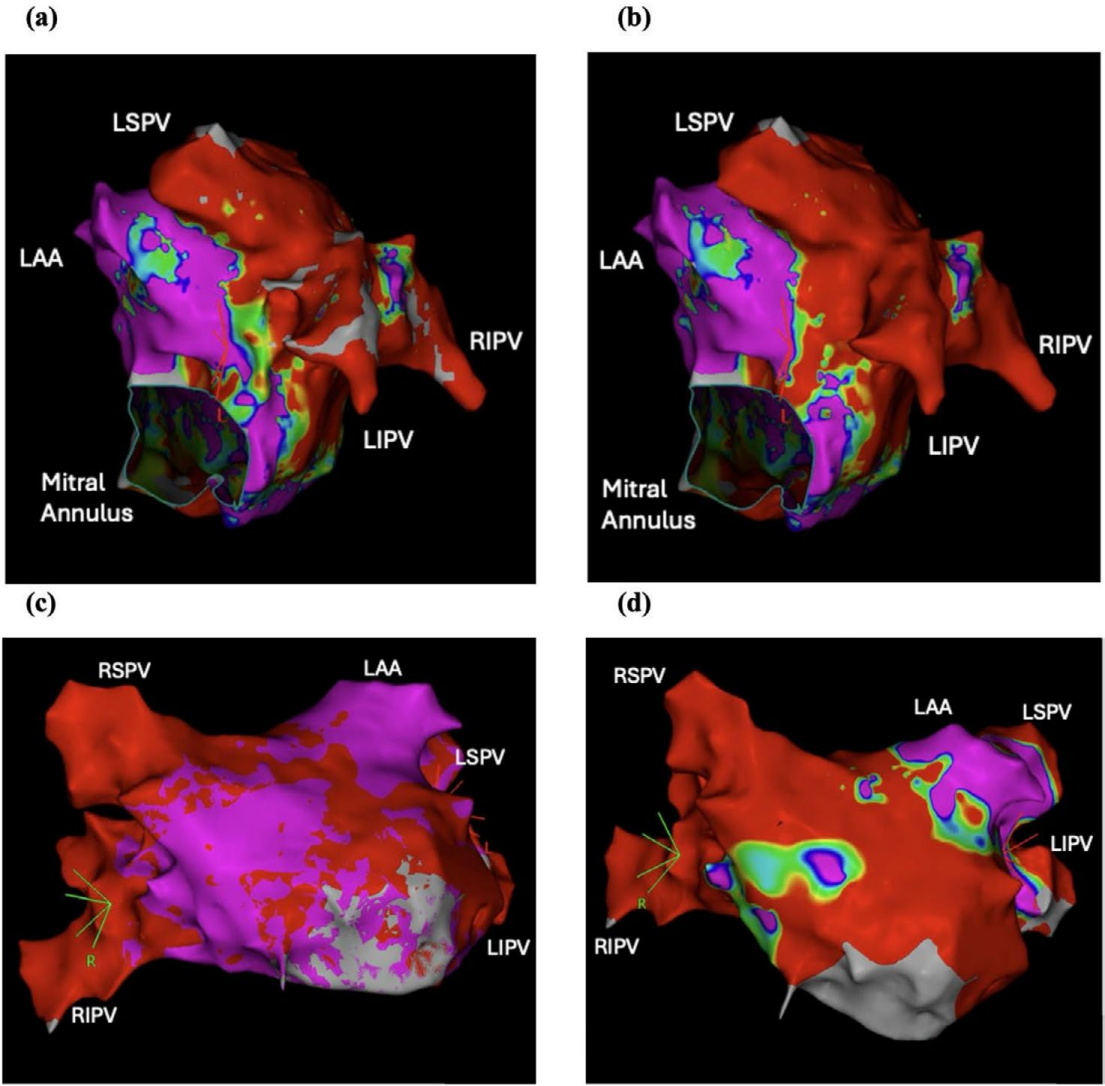
Voltage maps of anteroseptal and posterolateral mitral isthmus lines pre‐ and post‐ablation. 3D Electroanatomic map showing voltage scar pre‐ablation and post‐ablation along the posterolateral mitral isthmus (a, b) and anteroseptal mitral isthmus line (c, d). LAA, left atrial appendage; LIPV, left inferior pulmonary vein; LSPV, left superior pulmonary vein; RIPV, right inferior pulmonary vein; RSPV, right superior pulmonary vein.

For cases requiring additional ablation, the LAPW post‐ablation remap showed 3 early reconnections 3/55, 5.5% reconnection rate, while MI line reconnection was 6/14, 30% reconnection rate (Table 5). The anterior MI line reconnected in 3/7 cases (57%) while the posterior MI line reconnected in 2/13 cases (15%) (Figure 4). In all cases, the LAPW and MI line were isolated at the end of the procedure as confirmed by 3D EAM. There was no significant difference in arrhythmia recurrence at 12 months if reconnection was observed on immediate remap for the PVs and LAPW; however early reconnection of the MI line was associated with arrhythmia recurrence (4/16 cases with recurrence 25% vs. 3/58 cases without recurrence 2.3%, *p* = 0.02). In 3 cases a switch to RFA was required, 2 to perform a linear ablation line of cavo‐triscuspid isthmus and 1 to perform ablation of the superior vena cava as AF recurred within the right atrium. In all 3 cases, AF or AFL terminated on switch to RFA ablation but was not associated with a reduction in arrhythmia recurrence (1/16 patients with recurrence, 6.3% vs. 2/58 patients without recurrence 3.4%, *p* = 0.17).

**TABLE 5:** Acute durability for pulmonary vein isolation, mitral isthmus line and posterior wall isolation post pulsed field ablation.

Anatomical ablation	*n*	*n* (%)
Pulmonary vein isolation	296	289 (98)
LSPV	74	74 (100)
LIPV	74	73 (98)
RSPV	74	68 (92)
RIPV	74	74 (100)
Posterior wall isolation	55	52/55 (95)
Mitral isthmus	20	14/20 (70)
Anterior	7	3/7 (43)
Posterior	13	11/13 (85)

Abbreviations: LIPV, left inferior pulmonary vein; LSPV, left superior pulmonary vein; RIPV, right inferior pulmonary vein; RSPV, right superior pulmonary vein.

### 3.6 Pulsed Field Ablation Scar Border Analysis

**FIGURE 5:**
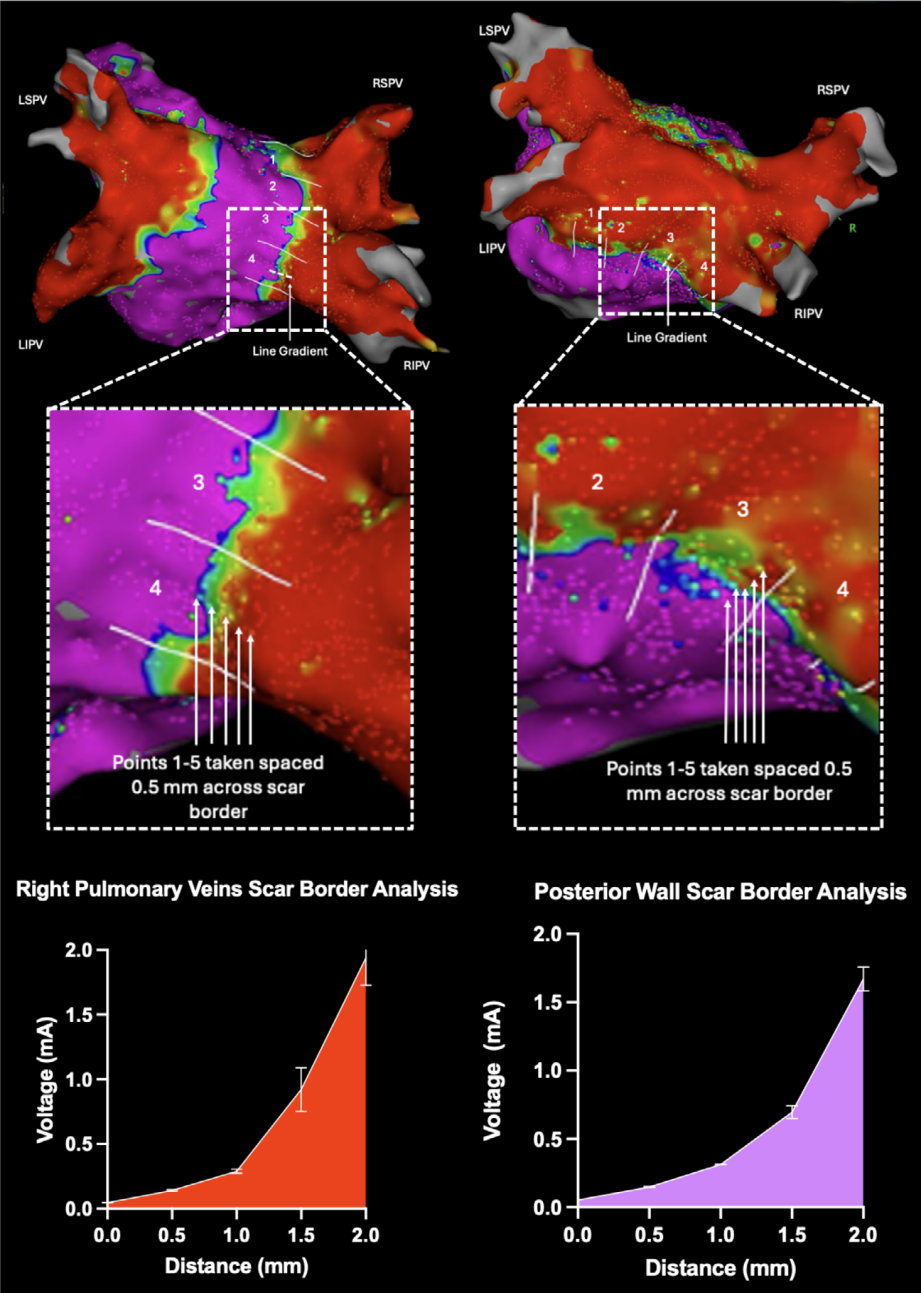
Graph of the scar border transition zone along pulsed field ablation lesion sets.

Analysis of the PFA scar border showed an average 1 mm distance between areas of normal voltage and low‐voltage areas. A significant drop in voltage across a 1 mm distance was observed from 1.94 ± 1.20 mV to 0.29 ± 0.9 mV across the right PVs (*p* < 0.001) and from 1.67 ± 0.37 mV to 0.31 ± 0.01 mV (*p* < 0.001) across the LAPW. Figure 5 shows the method for analysis of the PFA scar border and line graphs showing the scar border transition zone across the right PVs and LAPW.

### 3.7 Redo Procedures and Durability of Pulsed Field Ablation

Four patients underwent redo procedures at a mean interval of 237.5 ± 97.0 days. Detailed characteristics are shown in Table S1. All four patients were classified as persistent AF at the time of the first procedure. At redo mapping, PV reconnection was observed in 3/4 cases (75%), all involving right‐sided PVs: one case had both RSPV and LSPV reconnection, one had isolated RSPV reconnection, and one had isolated RIPV reconnection. The remaining case maintained complete PVI but demonstrated LAPW reconnection. The anterior MI line performed in one patient remained durably isolated at redo. The predominance of right‐sided PV reconnections at redo (3/4 cases, 100% of PV reconnections) is consistent with our acute remapping findings, where 6/7 acute reconnections occurred in the RSPV. This pattern suggests that right‐sided PVs, particularly the RSPV, represent anatomically challenging targets for PFA, likely related to the anterior‐superior aspect of the ostium, where catheter‐tissue contact may be suboptimal.

## 4 Discussion

The major findings of this study are:

1. PFA is clinically effective with 78% of patients clinically free of arrhythmia at 12 months as assessed by real‐world clinical follow‐up (without continuous monitoring from an implantable loop recorder or prolonged non‐invasive monitoring) upon integrating PFA into our hospital workflow.
2. PFA appears to be relatively safe with only one complication in our study of cardiac tamponade, which occurred in a case where a switch to RFA occurred.
3. 3D EAM was effective in showing an acute PV reconnection rate in 9% of patients.
4. MI line ablation was challenging using PFA with a 30% acute reconduction rate, which was significantly higher among anteroseptal lines (57%) versus posterolateral lines (15%).
5. PFA shows distinct lesion margins in a novel assessment of change in voltage across LA voltage EAM lesion borders.

### 4.1 Real World Application of Pulsed Field Ablation

Our data show that PFA is an effective treatment for AF ablation with 78% of patients' arrhythmia‐free at 12 months comparable to international data from the EU‐PORIA registry for PFA (74%) and REAL‐AF for RFA (81%) [9, 10]. Procedure times and fluoroscopy times were 48–129 min and 4–20 min, respectively. Our fluoroscopy time is reported as pedal time, but we used the lowest fluoro rate of 3 pulses per second in all cases. This is considerably shorter than procedure times typically reported for RFA in European centers, which average 82–128 min for cryoablation and 140–162 min for point‐by‐point RFA [11, 12]. In the ADVENT Trial, a randomized control trial proving that PFA was non‐inferior to RFA with respect to freedom from atrial arrhythmia at 12 months but faster in terms of procedure time (106 min vs. 123 min) and LA dwell time (59 min vs. 84 min) but not fluoroscopy time (21 min vs. 14 min) [6]. One potential explanation for this is the widespread use of 3D EAM in the United States (US) compared to the adaptation of PFA in Europe, where only a third of operators in the EU‐PORIA registry used 3D EAM [9, 10]. Our fluoroscopy times (12.1 ± 8.0 min) compare favorably to the ADVENT Trial, where the mean fluoroscopy time was 21 min for PFA versus 14 min for RFA. The EU‐PORIA registry reported fluoroscopy times ranging from 4 to 20 min. It is important to note that fluoroscopy in our protocol was primarily utilized for transseptal puncture (dual‐plane fluoroscopy and contrast guidance), catheter exchanges, and coronary sinus catheter placement—procedures that are necessary regardless of whether 3D mapping is performed. The 3D mapping itself is performed entirely without fluoroscopy.

### 4.2 Safety and Complications

Our data would suggest that PFA is safe with only one recorded complication in our cohort study of pericardial tamponade. This occurred in one patient where the PFA catheter was exchanged for an RFA catheter in the RA to perform a CTI line. PFA applications were initially delivered to the CTI; however, bidirectional block was not achieved, and an RFA catheter was used to complete the CTI line. Although not apparent on the ECG, it is possible that subclinical spasm of the right coronary artery could have been induced by the delivery of PFA to the CTI region triggering ischaemia, and subsequent RF applications to the local ischaemic tissue could have contributed to the development of tamponade. Coronary vasospasm has been observed with PFA in 41% during MI line ablation and 80% during CTI line ablation, while cardiac tamponade has been reported in 1% of cases [6, 13, 14, 15]. Boluses of intravenous nitroglycerin have been shown to prevent right coronary vasospasm during PFA ablation; however, that paper was published after our complication had occurred [14]. Our finding raises questions about the safety of using RFA directly after PFA without nitroglycerin in the right atrial isthmus.

### 4.3 The Clinical Utility of 3D Electroanatomical Mapping

Use of 3D EAM in our cohort revealed acute reconnection rates of 9% for the PVs, 5.5% for the LAPW, and 30% for the MI line. All PVs were isolated at the end of the procedure, and statistically, acute reconnection of the PVs was not associated with recurrence. This finding differs from previously published data showing first‐pass PFA PVI rates of 99.9%–100% [2, 7, 9]. The discordance between our findings and published first‐pass isolation rates may be explained by differences in assessment methodology. After the appearance of isolation on mapping with the Farawave catheter, the detection of ongoing conduction or acute reconnection by a flexible multisplined mapping catheter (Octaray) with small electrodes, small inter‐electrode spacing, and flexible independent splines may arise for two reasons: (1) poor suitability of the Farawave ablation catheter for detailed mapping due to its large electrodes (8 mm) and wide inter‐electrode spacing, or (2) the approximately two‐minute delay involved with catheter exchange allowing time for acute reconnection. The former explanation seems more likely given the inherent mapping limitations of the PFA catheter design.

The question of whether 3D mapping provides added value in PFA workflows must be considered in the context of current catheter technology limitations and study design. Our study was designed to evaluate the real‐world utility of 3D mapping in identifying acute reconnections during PFA procedures rather than to demonstrate superiority over non‐mapping approaches through a randomized controlled design. The Farawave PFA catheter's large electrodes and wide inter‐electrode spacing make it inherently unsuited for detailed mapping and isolation confirmation. In our experience, this resulted in a 9% discordance rate between PFA catheter assessment and high‐density mapping. While a randomized trial would be ideal to definitively establish the impact of systematic remapping on long‐term outcomes, the current limitations of PFA catheter mapping capability, combined with our observation that MI reconnection significantly predicted recurrence (*p* = 0.02), support the continued use of systematic 3D remapping protocols. Importantly, all seven reconnected PVs identified by 3D mapping were successfully re‐isolated, and while acute PV reconnection did not significantly predict long‐term recurrence (likely because they were successfully re‐isolated), mitral isthmus reconnection was significantly associated with arrhythmia recurrence (*p* = 0.02), supporting the clinical value of systematic remapping.

Interestingly, recent follow‐up data from the EU‐PORIA registry found that concomitant use of 3D EAM was not associated with PV durability (31.5% in those without PV reconnection vs. 36.7% in those with PV reconnection, *p* = 0.651) [16]. However, the impact of incomplete or acute PV reconnection identification by 3D EAM can only be fully assessed in a randomized controlled trial using systematic remapping protocols. Future development of PFA catheters with integrated high‐density mapping capabilities may obviate the need for separate mapping catheters. Pooled data from IMPULSE, PEFCAT, and PEFCAT II trials showed that PV reconnections at 2–3 months were observed in the superior veins [2]. Further data from Gunawardene et al. of 20 patients found that acute PV reconnection occurred in 5/80 PVs, all in the superior PVs (2 RSPV, 3 LSPV) with gaps primarily located in the anterior‐superior PV ostia [17]. This is consistent with our findings that acute PV reconnections occur primarily in the superior veins, particularly in the anterior‐superior PV ostia (Figure 2). In contrast, the EU‐PORIA registry found that left and right‐sided PVs had similar durability rates (RSPV 76%, LIPV 72%, RIPV 69%, LSPV 68%), though patients with a left common pathway showed poor lesion durability (44%) [16].

The LAPW showed acute reconnection in 5.5% of cases, most commonly along the inferior wall. Limited data are available on acute reconnection rates for LAPW isolation. The PersAFOne study included a protocol‐mandated invasive remapping procedure at 3 months, which showed durable isolation in 21/21 LAPW cases [2]. One retrospective analysis showed that the addition of PW isolation to standard PVI had no effect on arrhythmia recurrence at 1 year [18].

### 4.4 The Use of Pulsed Field Ablation on the Mitral Isthmus

Our data on MI line ablation using PFA suggests that achieving bidirectional block is challenging, particularly regarding an anterior approach. Early reconnection or incomplete isolation on remap was 57% using an anterior approach and 15% with a posterior approach. In contrast, RFA of the MI line shows similar success rates between the anterior and posterior approaches [19]. The anterior approach has the advantage of shorter procedure duration without the need for coronary sinus ablation. The data available in the literature regarding MI line ablation using PFA is limited. One study of 45 patients with persistent AF, using a pentaspline PFA catheter in the flower position to extend an MI line from the mitral annulus to the LIPV found an acute reconnection rate of 13.3% (6/45 patients) using this posterior approach [20]. In this prospective cohort study, 2 patients experienced coronary vasospasm, while we did not experience clinical or electrocardiographic evidence of vasospasm with MI ablation using PFA. Diagnostic angiography of patients undergoing MI line ablation observed coronary vasospasm in 10/23 patients (43.5%), most of which were subclinical episodes of spasm (9/10 patients, 90%) [13].

### 4.5 Examination of Pulsed Field Ablation Lesion Borders

A novel analysis of the PFA scar border transition zone was performed in this study, which showed a sudden and acute change in voltage from healthy to areas of low voltage scar across a sharp margin of 1 mm for both the right PVs and LAPW. Pre‐clinical studies with histological analysis of porcine specimens show PFA in the LA and RA displays sharp margins with a clear demarcation between healthy and scar tissue [21, 22]. In comparison, RFA ablation lesions show margins of contraction necrosis 0.3–0.7 mm wide between healthy and necrotic tissue when applied to healthy myocardium, whereas when applied to scar tissue, the lesions have no distinct structure or demarcation [23]. PFA in scarred myocardium on the other hand, produced uniform and well‐demarcated lesions. Significant differences in voltage were noted at 0.5 mm and 1.0 mm across the lesion border in both the right PVs and LAPW. At a 1 mm distance, the mean voltage dropped from normal to a low‐voltage area; however, there was also a significant drop in voltage at 0.5 mm halfway along this gradient. This 0.5 mm difference doesn't quite meet the definition for a low‐voltage area (< 0.5 mV), but may represent the border zone of reversible electroporation, which may be created with PFA lesions around the core zone of irreversible electroporation [24]. While we observed sharp voltage transitions at the PFA lesion border (mean 1 mm transition zone), we found no consistent correlation between voltage gradient characteristics and sites of acute reconnection in our cohort. This may reflect the small number of reconnection events (*n* = 7) or suggest that acute reconnections are primarily related to anatomical factors or incomplete lesion delivery rather than lesion quality variations. The clinical significance of our scar border analysis requires further investigation in larger cohorts. We included this analysis as a descriptive characterization of PFA lesion morphology on voltage mapping, which has not been previously reported in detail in the clinical literature. Larger studies would be needed to determine whether voltage gradient analysis can predict areas at risk for reconnection.

### 4.6 Limitations

Firstly, this is a retrospective, observational, and non‐randomized study with no control arm detailing our experience with incorporating PFA into our catheter ablation workflow. Secondly, this is a single‐center study with a single operator experience using PFA which creates a potential bias in patient selection and clinical management. Thirdly, the limited sample size in a tertiary referral center using one PFA system with one 3D EAM system may impair the generalizability of the study and limit the assessment of complications. Fourthly, some episodes of asymptomatic recurrent AF may have been missed as continuous invasive monitoring was not utilized. Fourthly, our follow‐up was limited to one year, so comparatively longer outcomes are not known. However, recurrence of symptomatic AF appeared infrequently at 2–5 years in a single‐center study of paroxysmal AF [25]. Finally, the absence of a control group (PFA without 3D mapping) limits our ability to definitively prove that 3D mapping improves long‐term outcomes, though our data demonstrate its utility in identifying and enabling treatment of acute reconnections. A randomized controlled trial comparing PFA with and without systematic 3D remapping would be needed to definitively establish whether identification and treatment of acute reconnections improve long‐term clinical outcomes. The small number of redo procedures (*n* = 4) limits our ability to correlate initial scar morphology with chronic reconnection patterns or to draw definitive conclusions about anatomical predictors of reconnection.

## 5 Conclusion

In conclusion, early PV reconnection or incomplete PV isolation can occur using PFA for PVI in almost one out of ten cases. The use of 3D EAM resulted in the successful isolation of all PVs in all cases demonstrating the enduring clinical utility of 3D EAM in AF ablation. The introduction of PFA to our center resulted in similar success rates, a low complication rate, and faster procedure times compared to conventional RFA.

## Funding

The authors have nothing to report.

## Ethics Statement

Institutional review board ethics approval was sought and attained.

## Conflicts of Interest

The authors declare no conflicts of interest.

## Supporting information


**Supplementary Table 1.** Summary of four re‐do cases post pulsed field ablation.

## References

[1] J. A. Joglar , M. K. Chung , A. L. Armbruster , et al., “2023 ACC/AHA/ACCP/HRS Guideline for the Diagnosis and Management of Atrial Fibrillation: A Report of the American College of Cardiology/American Heart Association Joint Committee on Clinical Practice Guidelines,” Circulation 149, no. 1 (2024): e1–e156. 38033089 10.1161/CIR.0000000000001193 PMC11095842

[2] V. Y. Reddy , A. Anic , J. Koruth , et al., “Pulsed Field Ablation in Patients With Persistent Atrial Fibrillation,” Journal of the American College of Cardiology 76, no. 9 (2020): 1068–1080. 32854842 10.1016/j.jacc.2020.07.007

[3] A. Sugrue , E. Maor , F. Del‐Carpio Munoz , A. M. Killu , and S. J. Asirvatham , “Cardiac Ablation With Pulsed Electric Fields: Principles and Biophysics,” Europace 24, no. 8 (2022): 1213–1222. 35426908 10.1093/europace/euac033

[4] S. Avazzadeh , B. O'Brien , K. Coffey , M. O'Halloran , D. Keane , and L. R. Quinlan , “Establishing Irreversible Electroporation Electric Field Potential Threshold in A Suspension In Vitro Model for Cardiac and Neuronal Cells,” Journal of Clinical Medicine 10, no. 22 (2021): 5443. 34830725 10.3390/jcm10225443 PMC8622402

[5] S. Avazzadeh , M. H. Dehkordi , P. Owens , et al., “Establishing Electroporation Thresholds for Targeted Cell Specific Cardiac Ablation in a 2D Culture Model,” Journal of Cardiovascular Electrophysiology 33, no. 9 (2022): 2050–2061. 35924470 10.1111/jce.15641 PMC9543844

[6] V. Y. Reddy , E. P. Gerstenfeld , A. Natale , et al., “Pulsed Field or Conventional Thermal Ablation for Paroxysmal Atrial Fibrillation,” New England Journal of Medicine 389, no. 18 (2023): 1660–1671. 37634148 10.1056/NEJMoa2307291

[7] M. K. Turagam , P. Neuzil , B. Schmidt , et al., “Safety and Effectiveness of Pulsed Field Ablation to Treat Atrial Fibrillation: One‐Year Outcomes From the MANIFEST‐PF Registry,” Circulation 148, no. 1 (2023): 35–46. 37199171 10.1161/CIRCULATIONAHA.123.064959

[8] E. Ekanem , P. Neuzil , T. Reichlin , et al., “Safety of Pulsed Field Ablation in More Than 17,000 Patients With Atrial Fibrillation in the MANIFEST‐17K Study,” Nature Medicine 30, no. 7 (2024): 2020–2029. 10.1038/s41591-024-03114-3 PMC11271404 38977913

[9] B. Schmidt , S. Bordignon , K. Neven , et al., “EUropean Real‐World Outcomes With Pulsed Field AblatiOn in Patients With Symptomatic atRIAl Fibrillation: Lessons From the Multi‐Centre EU‐PORIA Registry,” Europace 25, no. 7 (2023), 10.1093/europace/euad185. PMC10320231 37379528

[10] J. Osorio , A. F. Miranda‐Arboleda , A. Velasco , et al., “Real‐World Data of Radiofrequency Catheter Ablation in Paroxysmal Atrial Fibrillation: Short‐ and Long‐Term Clinical Outcomes From the Prospective Multicenter REAL‐AF Registry,” Heart Rhythm 21 (2024): 2083–2091. 38768839 10.1016/j.hrthm.2024.04.090

[11] K. H. Kuck , J. Brugada , A. Fürnkranz , et al., “Cryoballoon or Radiofrequency Ablation for Paroxysmal Atrial Fibrillation,” New England Journal of Medicine 374, no. 23 (2016): 2235–2245. 27042964 10.1056/NEJMoa1602014

[12] E. Arbelo , J. Brugada , C. Blomström‐Lundqvist , et al., “Contemporary Management of Patients Undergoing Atrial Fibrillation Ablation: In‐Hospital and 1‐Year Follow‐Up Findings From the ESC‐EHRA Atrial Fibrillation Ablation Long‐Term Registry,” European Heart Journal 38, no. 17 (2017): 1303–1316. 28104790 10.1093/eurheartj/ehw564

[13] C. Zhang , P. Neuzil , J. Petru , et al., “Coronary Artery Spasm During Pulsed Field vs Radiofrequency Catheter Ablation of the Mitral Isthmus,” JAMA Cardiology 9, no. 1 (2024): 72–77. 38019505 10.1001/jamacardio.2023.4405 PMC10687713

[14] Y. Malyshev , P. Neuzil , J. Petru , et al., “Nitroglycerin to Ameliorate Coronary Artery Spasm During Focal Pulsed‐Field Ablation for Atrial Fibrillation,” JACC: Clinical Electrophysiology 10, no. 5 (2024): 885–896. 38385916 10.1016/j.jacep.2023.12.015

[15] T. Deneke , K. Nentwich , R. Schmitt , et al., “Exchanging Catheters Over a Single Transseptal Sheath During Left Atrial Ablation Is Associated With a Higher Risk for Silent Cerebral Events,” Indian Pacing and Electrophysiology Journal 14, no. 5 (2014): 240–249. 25408564 10.1016/s0972-6292(16)30795-1 PMC4217296

[16] T. Kueffer , S. Bordignon , K. Neven , et al., “Durability of Pulmonary Vein Isolation Using Pulsed‐Field Ablation: Results From the Multicenter EU‐PORIA Registry,” JACC: Clinical Electrophysiology 10, no. 4 (2024): 698–708. 38340118 10.1016/j.jacep.2023.11.026

[17] M. A. Gunawardene , B. N. Schaeffer , M. Jularic , et al., “Pulsed‐Field Ablation Combined With Ultrahigh‐Density Mapping in Patients Undergoing Catheter Ablation for Atrial Fibrillation: Practical and Electrophysiological Considerations,” Journal of Cardiovascular Electrophysiology 33, no. 3 (2022): 345–356. 34978360 10.1111/jce.15349

[18] M. K. Turagam , P. Neuzil , B. Schmidt , et al., “Impact of Left Atrial Posterior Wall Ablation During Pulsed‐Field Ablation for Persistent Atrial Fibrillation,” JACC: Clinical Electrophysiology 10, no. 5 (2024): 900–912. 38430087 10.1016/j.jacep.2024.01.017

[19] M. Huemer , A. Wutzler , A. S. Parwani , et al., “Comparison of the Anterior and Posterior Mitral Isthmus Ablation Lines in Patients With Perimitral Annulus Flutter or Persistent Atrial Fibrillation,” Journal of Interventional Cardiac Electrophysiology 44, no. 2 (2015): 119–129. 26129787 10.1007/s10840-015-0033-1

[20] B. Davong , R. Adeliño , H. Delasnerie , et al., “Pulsed‐Field Ablation on Mitral Isthmus in Persistent Atrial Fibrillation: Preliminary Data on Efficacy and Safety,” JACC: Clinical Electrophysiology 9, no. 7, Part 2 (2023): 1070–1081. 37354173 10.1016/j.jacep.2023.03.021

[21] J. Koruth , K. Kuroki , J. Iwasawa , et al., “Preclinical Evaluation of Pulsed Field Ablation,” Circulation. Arrhythmia and Electrophysiology 12, no. 12 (2019): e007781. 31826647 10.1161/CIRCEP.119.007781 PMC6924932

[22] M. Terricabras , R. P. Martins , R. Peinado , et al., “Cardiac Pulsed Field Ablation Lesion Durability Assessed by Polarization‐Sensitive Optical Coherence Reflectometry,” Circulation. Arrhythmia and Electrophysiology 17, no. 3 (2024): e012255. 38318720 10.1161/CIRCEP.123.012255 PMC10949975

[23] M. Barkagan , E. Leshem , A. Shapira‐Daniels , et al., “Histopathological Characterization of Radiofrequency Ablation in Ventricular Scar Tissue,” JACC: Clinical Electrophysiology 5, no. 8 (2019): 920–931. 31439293 10.1016/j.jacep.2019.05.011

[24] T. Kueffer , A. Stefanova , A. Madaffari , et al., “Pulmonary Vein Isolation Durability and Lesion Regression in Patients With Recurrent Arrhythmia After Pulsed‐Field Ablation,” Journal of Interventional Cardiac Electrophysiology 67, no. 3 (2024): 503–511. 37523023 10.1007/s10840-023-01608-7 PMC11015999

[25] D. R. Musikantow , P. Neuzil , A. Anic , et al., “Long‐Term Clinical Outcomes of Pulsed Field Ablation in the Treatment of Paroxysmal Atrial Fibrillation,” JACC: Clinical Electrophysiology 9, no. 9 (2023): 2001–2003.37565951

