# Supplementary material for: The Clinical Utility of 3D Electroanatomical Mapping for Atrial Fibrillation Ablation by Pulsed Field Ablation
Source: J Arrhythm. 2025 Nov 27;41(6):e70234. doi: 10.1002/joa3.70234 (PMC12661062; doi:10.1002/joa3.70234)
Supplement: Supplementary file 1 — Supplementary Table 1. Summary of four re‐do cases post pulsed field ablation. [file JOA3-41-e70234-s001.docx]

**Supplementary Table 1 Summary of four re-do cases post pulsed field ablation.**

| Case | Recurrent rhythm | Time to redo (days) | RSPV | RIPV | LSPV | LIPV | Further Ablation |
| --- | --- | --- | --- | --- | --- | --- | --- |
| 1 | AF | 170 | X |  | X |  |  |
| 2 | AF | 179 |  | X |  |  |  |
| 3 | AFL | 321 |  |  |  |  | LAPW reconnection |
| 4 | AFL | 330 |  |  |  |  | Anterior MI line remained isolated |

LSPV indicates, left superior pulmonary vein; RSPV, right superior pulmonary vein; LIPV, left inferior pulmonary vein; RIPV, right inferior pulmonary vein.
